# Supplementary material for: Contribution of Genetic Test to Early Diagnosis of Methylenetetrahydrofolate Reductase (MTHFR) Deficiency: The Experience of a Reference Center in Southern Italy
Source: Genes (Basel). 2023 Apr 26;14(5):980. doi: 10.3390/genes14050980 (PMC10218448; doi:10.3390/genes14050980)
Supplement: Supplementary file 1 [file genes-14-00980-s001.zip › genes-2327514-supplementary.pdf]

Table S1.Time-course of biomarkers and therapeutic regiment of four newborns with a positive newborn screen for MTHFR deficiency.

Patient 1

	DBS		*URINE*		*SERUM*									
	I	*II*	*4/20/18*	*11/7/18*	*3/7/18*	*11/7/18*	*2/5/19*	*5/30/19*	*10/29/19*	*3/3/20*	*10/6/20*	*4/19/21*	*4/19/22*	*9/27/22*
**Met**:<br>*cut-off DBS: 6-20 µmol/L*<br>*rv serum: 10-60 µmol/L (<1 month)*<br>*9-42 µmol/L (>1 month)*	4	6				10								26
**C3**:<br>*cut-off DBS: 3.18 µmol/L*<br>*rv serum: 0.04-3.18 µmol/L*	1.68					0.3								
**Hcy**:<br>*cut-off DBS: 4 µmol/L*<br>*rv serum: 5-15 µmol/L*	53.7	42.3			106.7	51.7	47.1	56.9	44.8	50.6	45.5	52.9	51.5	52.4
**MMA**:<br>*rv urinary: nd-5 µmol/L*			4	2										
**Maternal Vit B12**:<br>*rv serum: 197-866 pg/mL*														
**Vit B12**:<br>*rv serum: 197-866 pg/mL*								502	586		390		526	540
**Therapy***:<br>*M: mg/die*<br>*B12: mg/die (i.m.)*<br>*B: mg/kg/die*<br>*M: Mefolinate, B: Betaine						M: 45<br>B12: 1<br>B: 250	M: 45<br>-<br>B: 250	M: 45<br>-<br>B: 250	M: 45<br>-<br>B: 250	M: 45<br>-<br>B: 250	M: 45<br>-<br>B: 250	M: 45<br>-<br>B: 250	M: 60<br>-<br>B: 250	M: 60<br>-<br>B: 250

DBS: Dry Blood Spot; Met: Methionine; C3: C3-Carnitine; Hcy: Homocysteine; MMA: Methylmalonic Acid.

Patient 2

	DBS		*URINE*		*SERUM*	
	I	*II*	*3/9/20*	*5/28/20*	*3/9/20*	*5/19/20*
**Met**:<br>*cut-off DBS: 6-20 µmol/L*<br>*rv serum: 10-60 µmol/L (<1 month)*<br>*9-42 µmol/L (>1 month)*	5.5	9			41	31
**C3**:<br>*cut-off DBS: 3.18 µmol/L*<br>*rv serum: 0.04-3.18 µmol/L*	3.03				1.29	
**Hcy**:<br>*cut-off DBS: 4 µmol/L*<br>*rv serum: 5-15 µmol/L*	4.4	5.11			14.7	17.3
**MMA**:<br>*rv urinary: nd-5 µmol/L*			38	37		
**Maternal Vit B12**:<br>*rv serum: 197-866 pg/mL*					367	
**Vit B12**:<br>*rv serum: 197-866 pg/mL*						
**Therapy***:<br>*FA: µg/die*<br>*FA: Folic Acid						FA: 400

DBS: Dry Blood Spot; Met: Methionine; C3: C3-Carnitine; Hcy: Homocysteine; MMA: Methylmalonic Acid.

Patient 3

	DBS		*URINE*		*SERUM*									
	I	*II*	*12/2/20*	*3/25/21*	*11/4/20*	*12/2/20*	*1/22/21*	*3/25/21*	*6/15/21*	*9/21/21*	*12/10/21*	*3/23/22*	*7/22/22*	*11/4/22*
**Met**:<br>*cut-off DBS: 6-20 µmol/L*<br>*rv serum: 10-60 µmol/L (<1 month)*<br>*9-42 µmol/L (>1 month)*	5.07	8.6				29								
**C3**:<br>*cut-off DBS: 3.18 µmol/L*<br>*rv serum: 0.04-3.18 µmol/L*	0.89					0.7								
**Hcy**:<br>*cut-off DBS: 4 µmol/L*<br>*rv serum: 5-15 µmol/L*	11	16.3			53.1	41.9	33.6	28.9	30.3	26.5	28.2	25.0	27.9	28.8
**MMA**:<br>*rv urinary: nd-5 µmol/L*			5	5										
**Maternal Vit B12**:<br>*rv serum: 197-866 pg/mL*														
**Vit B12**:<br>*rv serum: 197-866 pg/mL*						347	371	554	356	271	224	>2000	>2000	>2000
**Therapy***:<br>*M: mg/die*<br>*B12: mg/die (o.s.)*<br>*B: mg/kg/die*<br>*M: Mefolinate, B: Betaine						M: 45<br>-<br>B:250				M: 45<br>B12: 0.33<br>B: 250				M: 45<br>B12: 0.33<br>B: 250

DBS: Dry Blood Spot; Met: Methionine; C3: C3-Carnitine; Hcy: Homocysteine; MMA: Methylmalonic Acid.

Patient 4

	DBS		*URINE*		*SERUM*	
	I	*II*	*8/4/22*	*9/8/22*	*8/4/22*	*9/8/22*
**Met**:<br>*cut-off DBS: 6-20 µmol/L*<br>*rv serum: 10-60 µmol/L (<1 month)*<br>*9-42 µmol/L (>1 month)*	5.6	8			23	
**C3**:<br>*cut-off DBS: 3.18 µmol/L*<br>*rv serum: 0.04-3.18 µmol/L*	3.02				1.5	0.3
**Hcy**:<br>*cut-off DBS: 4 µmol/L*<br>*rv serum: 5-15 µmol/L*	9.2	7			54.5	3.7
**MMA**:<br>*rv urinary: nd-5 µmol/L*			403	9		
**Maternal Vit B12**:<br>*rv serum: 197-866 pg/mL*					143	
**Vit B12**:<br>*rv serum: 197-866 pg/mL*					<83	>2000
**Therapy***:<br>*B12: mg/die (o.s. 6 days/week and i.m. once/week)*<br>*C: mg/kg/die*<br>*C: Carnitine					B12: 0.33<br>C: 100	

DBS: Dry Blood Spot; Met: Methionine; C3: C3-Carnitine; Hcy: Homocysteine; MMA: Methylmalonic Acid.
